# Epidemiological Profile, Clinical Presentations, and Complications of Gunshot Wounds in Patients Admitted to a Tertiary Hospital in the Armed Conflict Zone of Goma in Eastern DR Congo

**DOI:** 10.1002/puh2.70274

**Published:** 2026-05-13

**Authors:** Samuel Mbabazi, Zubayer Shams, Phalek Mukaka, Luc Kinyoma, Reagan Luvande, Jones Onesime, Aymar Akilimali, Médard Kabuyaya

**Affiliations:** ^1^ Faculty of Medicine University of Goma Goma DR Congo; ^2^ Department of Surgery HEAL Africa Tertiary Hospital Goma DR Congo; ^3^ Brunel Medical School Brunel University London UK; ^4^ Epiter's Consulting Office DRC Global Health Research Units Kinshasa DR Congo; ^5^ Department of Research Medical Research Circle (MedReC) Goma DR Congo

**Keywords:** armed conflict, complications, Democratic Republic of Congo, gunshot wound, traumatology

## Abstract

**Background:**

Gunshot wounds are a major cause of mortality in armed conflict settings. In Democratic Republic of Congo (DRC), particularly in the east of the country, recurring armed violence results in a significant influx of wounded requiring emergency surgical care. This study aims to describe the epidemiological, clinical profile, and complications observed in patients with gunshot wounds.

**Methods:**

Retrospective descriptive study was conducted on 242 patients admitted between December 2024 and February 2025. Sociodemographic, clinical characteristics, and outcome data were analyzed using STATA 14 software. Associations between clinical variables and complications were explored at the significance level *p* < 0.05.

**Results:**

Patient records 242 were included in this study. The victims were predominantly male (74.4%) and young adults with a median age of 25 (range 18–34) years. Injuries mainly affected the lower (44.2%) and upper (42.2%) limbs. Open fractures (17.8%) and abdominal injuries (17.8%) were the injuries most frequently associated with complications. Hemodynamic instability on admission (44.1% of complicated cases) and an admission delay exceeding 8 h (53% of complicated cases) significantly increased the risk of complications. The mean length of hospital stay was 20 ± 16 days, and the overall mortality rate was 1.2%.

**Conclusion:**

Gunshot wounds in the city of Goma primarily affect young, active men who are frequently exposed to conflict zones. Complications are linked to initial instability, delays in admission, and the severity of the injuries. Strengthening the prehospital care system and surgical capacity remains essential to reducing mortality.

## Graphical abstract

Gunshot wounds in eastern Democratic Republic of Congo primarily affect young men, with limb injuries being the most common and complications associated with open fractures and abdominal trauma. Hemodynamic instability upon admission and a delay in treatment exceeding 8 h significantly increase the risk of complications, highlighting the need to strengthen prehospital care and surgical capacity, particularly in Goma.

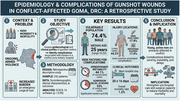

## 1 Introduction

Gun injuries are a major cause of mortality in conflict settings and a significant public health challenge in many resource‐limited regions. Globally, gun violence contributes substantially to the burden of trauma and disability‐adjusted life years (DALYs), with a marked geographical distribution and high prevalence in parts of sub‐Saharan Africa. These trends result in a high demand for emergency surgical care, resuscitation, and postoperative care needs that are often difficult to meet in fragile health systems [1].

The Democratic Republic of Congo (DRC), and more specifically the Kivu region in the east, remains one of the hotspots of protracted armed conflict in Africa. Clashes between armed groups, with their successive waves of hostilities over decades, have led to massive displacement, a deterioration of health infrastructure, and an increase in gun‐related injuries [2]. Recent reports from humanitarian and health organizations confirm a worsening humanitarian and health situation in North Kivu province and its surrounding areas, with direct consequences for the burden of serious injuries and the functioning of healthcare facilities [2]. Despite the clinical and social significance of gunshot wounds in this region, local scientific documentation remains limited. Available studies on the subject, conducted in referral hospitals or in neighboring settings, highlight specific epidemiological profiles: a predominance of male and young adult victims, a predominance of limb injuries, a high frequency of infectious complications, and persistent functional morbidity among survivors. These studies also underscore the critical impact of prehospital delays and hemodynamic status upon admission on clinical outcome (survival and sequelae) [3, 4].

Another determining factor in trauma outcomes in low‐ and middle‐income countries is the weakness of prehospital care systems. A notable observational study demonstrates that the absence of an organized first aid network, nonmedical transport, and delays in arrival at a facility capable of providing urgent surgical care are major contributors to preventable mortality. Improving trauma survival chains, rapid identification, early hemodynamic stabilization, and coordinated transfers to surgical centers is therefore a priority for reducing mortality and long‐term consequences in these settings [4, 5].

In this context, the study entitled “Epidemiological Profile and Clinical Characteristics of Patients with Gunshot Wounds at Heal Africa Hospital in Goma during the 2024‐Armed Conflict” aims to address several gaps: provide a detailed description of patients admitted for gunshot wounds during 2024; characterize the management modalities and complications observed; and identify the clinical and organizational factors associated with adverse outcomes. The expected results should inform hospital and humanitarian decision‐makers regarding priority interventions (strengthening prehospital care, optimizing surgical protocols, and enhancing resuscitation and appropriate antibiotic therapy capacities) likely to reduce morbidity and mortality related to gunshot wounds in this conflict context.

## 2 Methodology

### 2.1 Study Design and Setting

This is a retrospective descriptive study with an analytical aim carried out at the Heal Africa Hospital in Goma. It focused on the medical records of patients admitted for gunshot wounds between December 2024 and February 2025, a period marked by the armed conflict in the region.

Heal Africa Hospital is a tertiary care hospital located in Goma, North Kivu province, in the eastern DRC. It is recognized for its expertise in surgery, traumatology, and resuscitation and is a referral center for the care of victims of armed violence in eastern DRC.

### 2.2 Study Population

The study population consists of all patients hospitalized for gunshot wounds during the study period.

− Inclusion criteria: Any patient, regardless of age or sex, admitted for gunshot wounds and with a complete medical file.
− Exclusion criteria: Patients who are victims of other traumas (road traffic accidents, cuts, shrapnel, etc.) and incomplete or illegible records.

### 2.3 Sampling and Sample Size

An exhaustive sampling of all patients and records was used. All records meeting the inclusion criteria were retained for analysis.

### 2.4 Variables Studied

The variables collected included:

− Sociodemographic variables: age, sex, marital status, education level, and origin.
− Clinical variables: location of wounds, number of lesions, hemodynamic status on admission, and associated injuries (fractures, hemorrhages, and visceral damage).
− Therapeutic variables: time between trauma and admission.
− Evolving variables: length of hospital stays, complications, and outcome (recovery and death).

### 2.5 Source and Collection of Data

Data were extracted from the emergency room, operating room, and hospitalization records. A standardized data collection form was used to ensure the uniformity and reliability of the information gathered.

### 2.6 Operational Definitions

− **Gunshot wound**: any physical injury caused directly by the penetration of a firearm projectile, confirmed by clinical examination and/or imaging.
− **Admission time**: the time interval (in hours) between the occurrence of the trauma and the patient's arrival at Heal Africa Hospital.

- Early admission: ≤7 h.
- Late admission: >7 h.

− **Hemodynamic status on admission**:

- Stable: systolic blood pressure ≥90 mmHg and heart rate ≤100 beats/min.
- Unstable: systolic blood pressure <90 mmHg and/or heart rate >100 beats/min.

− **Surgical intervention**: any operative procedure (debridement, surgical exploration, laparotomy, bone fixation, etc.) performed to treat injuries related to trauma.
− **Complication**: any adverse event occurring during hospitalization related to the trauma or its management (infection, secondary hemorrhage, shock, etc.).
− **Patient outcome**:

- Recovery: discharged from hospital without any disabling after‐effects.
- Sequelae: persistence of functional deficits (paralysis, amputation, functional impairment, etc.) upon discharge.
- Death: death occurred during hospitalization.

### 2.7 Data Analysis

The data collected using KoboCollect was recorded in an Excel 2019 spreadsheet. After cleaning and checking for consistency and missing data, it was exported and analyzed using STATA 14 SE software.

− Quantitative variables were presented as mean ± standard deviation or median with interquartile range (IQR) according to their distribution.
− Qualitative variables were expressed as frequency and percentage.
− A bivariate analysis (Chi^2^ test or Fisher's exact test) was performed to explore the association between certain variables and outcomes (death and sequelae).
− The threshold for statistical significance was set at *p* < 0.05.

## 3 Results

### 3.1 Epidemiological Profile

Patients 242 were included, with a median age of 25 (range 18–34) years. The most affected age group was 20–29 years, representing 38.8% (94/242). Most victims were male (74.4%, 180/242). Most victims lived alone (82%, 192/242) and were unemployed (62%). Men and younger patients in the 20–29 age group presented with more complications and were unstable upon admission (Table 1).

**TABLE 1:** Epidemiological profile of gunshot wound patients at the Heal Africa Hospital in Goma in January 2025.

Variables	Total *n* = 242 (%)	Complications *n* = 34 (%)
**Sex**		
Man	180(74.4)	28(82.4)
Women	62(25.6)	06(17.6)
**Age range**		
<10 years old	23(9.5)	01(2.9)
10–19 years old	48(19.8)	05(14.7)
20–29 years old	94(38.8)	17(50)
30–39 years old	36(14.9)	06(17.7)
≥40 years old	41(16.9)	05(14.7)
**Marital status**		
Living alone	198(82)	26(76.5)
In a relationship/Union	44(18)	08(23.5)
**Level of education**		
No level	29(11.9)	02(5.9)
Primary	69(28.5)	14(41.2)
Secondary	106(43.8)	14(41.2)
Higher education/University	38(15.7)	04(11.8)
**Occupation**		
Unemployed	150(62)	16(47)
Employee	92(38)	18(53)

*Note:* n: sample size; (%): percentage.

### 3.2 Patient Origin

**FIGURE 1:**
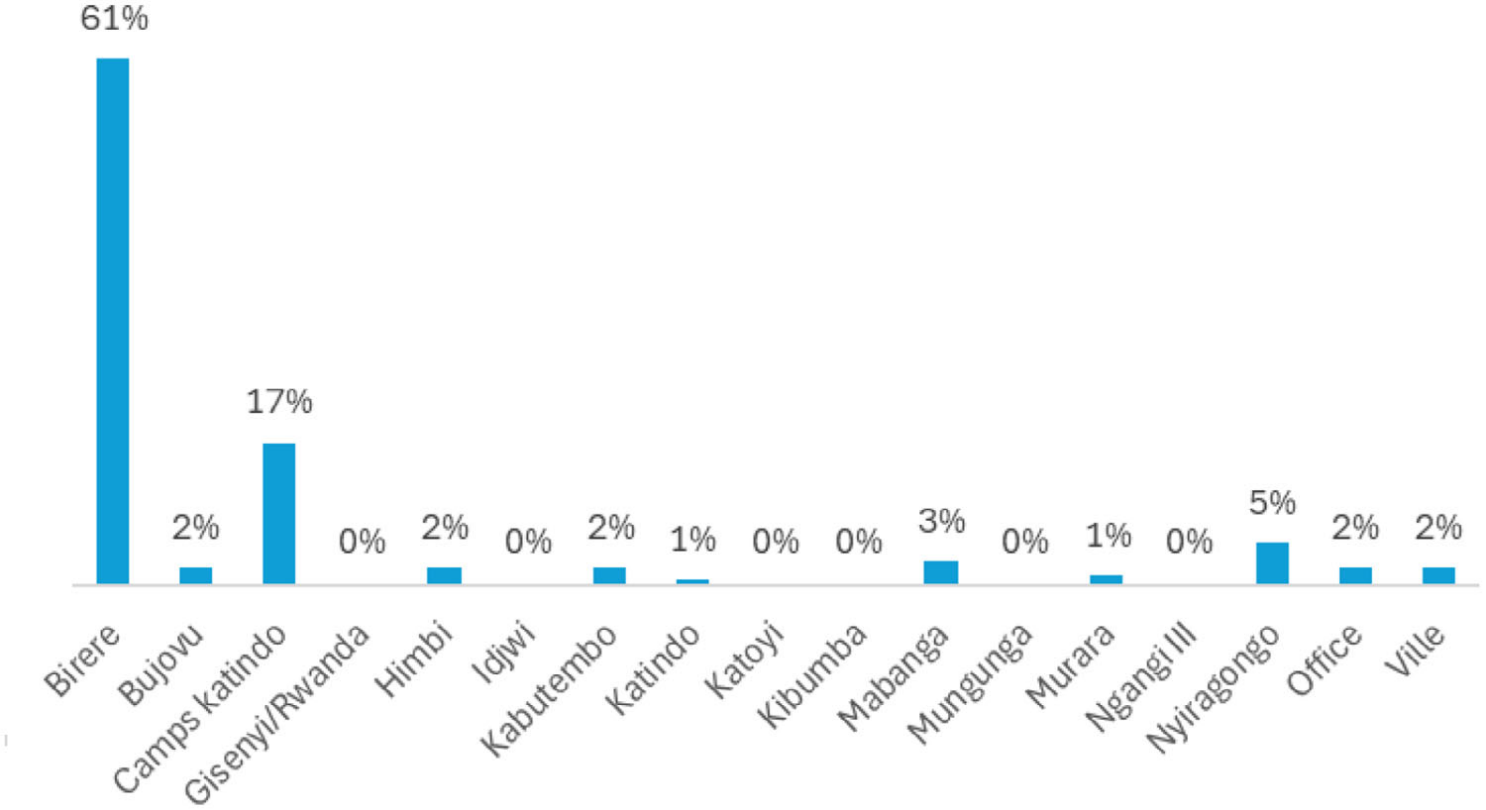
Distribution of patients according to their origin.

The majority (61%) of patients came from Birere, which is a peripheral area of the city of Goma and directly affected by the clashes (Figure 1).

### 3.3 Clinical Profile

On admission, nearly 86% (207/242) of the victims were hemodynamically stable. The most frequent injuries involved the lower limbs (44.2%) and upper limbs (42.2%). Open fractures (17.8%) and abdominal injuries (17.8%) were the most frequently associated with complications. The median time to admission was 6 h, with an IQR of 3–12. And more than half (65%) (157/242) of the victims were admitted within 8 h. The mean length of hospital stay was 20 ± 16 days, with prolonged stays (≥20 days) in 85.3% of complicated cases (Table 2).

**TABLE 2:** Clinical profile of gunshot patients admitted at Heal Africa Hospital, from December 2024 to January 2025.

Variables	Total *n* = 242 (%)	Complications *n* = 34 (%)
**Clinical condition upon arrival**		
Stable	207(85.5)	19(55.9)
Unstable	35(14.5)	15(44.1)
Presence of foreign bodies	88(36.4)	13(38.2)
**Site of the injury**		
Lower limbs	107(44.2)	13(38.2)
Upper limbs	102(42.2)	14(41.2)
Abdomen	43(17.8)	14(41.2)
Thorax	43(17.8)	07(20.6)
Neck	09(3.7)	01(03)
Head	22(9.1)	03(09)
**Nature of the trauma**		
Traumatic amputation	04(1.7)	01(03)
Internal organ perforation	17(07)	09(26.5)
Open fracture	43(17.8)	20(58.8)
Multiple penetrating wound	57(23.6)	08(23.5)
Single penetrating wound	130(53.7)	02(5.9)
**Hospital admission time in hours**	Median: 06(3–12) h	
≤8	157(65)	16(47)
>8	85(35.1)	18(53)
**Length of hospital stay in days**	Average: 20 ± 16 days	
<20	139(57.4)	05(14.7)
≥20	103(42.6)	29(85.3)
**Originating**		
Cured	239(99)	31(92)
Deceased	03(1.2)	03(8.8)

### 3.4 Issue

**FIGURE 2:**
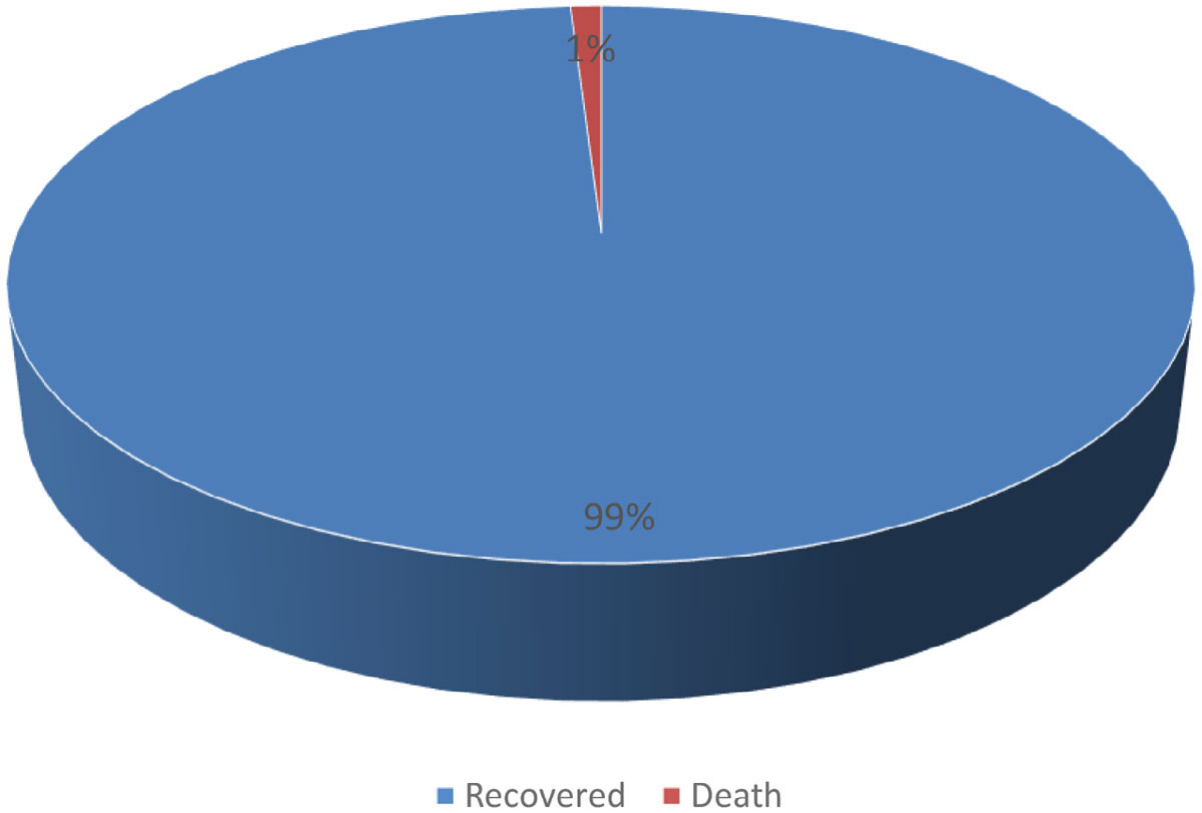
Outcome of gunshot wound patients at Heal Africa Hospital.

Almost all patients (99%) had a favorable outcome, whereas the mortality rate was low (1.2%) (Figure 2).

A comparison of outcomes according to injury burden demonstrated that patients with multiple injuries experienced a substantially higher frequency of complications than those with single injuries. Most complications occurred in patients presenting with open fractures and abdominal injuries, which were more common in the multi‑injury group. These patients also accounted for most prolonged hospitalizations (≥20 days), observed in 85.3% of complicated cases with an overall mortality rate of 1.2%. These findings indicate that sustaining multiple injuries was strongly associated with increased complication rates.

## 4 Discussion

The present study analyzes the epidemiological, clinical profile, and complications related to gunshot wounds in a tertiary hospital in the armed conflict zone of the DRC. The typical profile of gunshot wound victims in Goma: young, unemployed men, often living alone, and exposed to conflict environments. These findings corroborate those reported in Nigeria and East Africa, where most of the gunshot wound victims are young adult males [6, 7]. Limb injuries are the most frequent, but abdominal injuries and open fractures are the main sources of complications, due to the high risk of infection and surgical complexity. Similar studies in Ethiopia and South Sudan have also shown that visceral and bone trauma were the most severe [8, 9]. Prolonged admission delays (>8 h) are a major factor contributing to complications. This is due to the lack of a medical evacuation system and road insecurity, which limits rapid transfers. These constraints are also described in the reports by Doctors Without Borders and the World Health Organization in North Kivu [10, 11, 12, 13].

Despite the challenging conditions, the overall mortality rate (1.2%) remains relatively low, reflecting the surgical expertise of the region's tertiary hospital (Heal Africa). However, the specific mortality rate among complicated cases (8.8%) underscores the severity of the injuries and the need to strengthen postoperative care. Infections, such as abscesses, osteomyelitis, tetanus, sepsis, and gas gangrene, affect 20%–30% of patients due to wound contamination from environmental debris and bacteria during prolonged field exposure exceeding 12 h. Inadequate debridement upon arrival worsens anaerobic colonization, with postoperative rates reaching 15%–25% [14, 15]. In clinical context, hemorrhagic shock occurs in 30%–50% of patients as a direct result of vascular disruption and cavitation [16]. The severity of this shock is often compounded by blood loss during transport, preexisting anemia, and the scarcity of available transfusions [17]. Beyond the immediate circulatory collapse, extensive tissue damage frequently precipitates compartment syndrome, with fractures complicating 40%–70% of cases involving the extremities. Furthermore, organ perforation often leads to peritonitis or pneumothorax that frequently necessitates invasive measures such as mechanical ventilation or amputation [18].

Fatal outcomes are primarily driven by sepsis (35%), exsanguination (25%), and multi‐organ failure (20%). These complications correlate strongly with admission delays exceeding 24 h, which can extend hospital stays by 5–7 weeks and elevate mortality rates to 25%–40%. At the point of late arrival, a shock index greater than 1.0 and hemoglobin levels below 7 g/dL are potent predictors of a threefold increase in fatality risk. Conversely, early intervention within the first 6 h of injury has been shown to halve the risk of death through prompt medical and surgical management [19].

To add on, Budema et al. present a study from Heal Africa Hospital in Goma, DRC, which is highly relevant as it explores gunshot wound complications (29.1% overall rate) and their direct link to admission delays (mean 4.9 days), showing increased mortality risk (odds ratio, OR = 1.8), longer hospital stays (63 vs. 25 days), and higher infection/anemia rates. It explicitly recommends further statistical analysis of delay‐related fatal outcomes using delay frameworks [19].

### 4.1 Limitations of the Study

This study has the following limitations: The retrospective nature of the study exposes it to information bias, and the lack of posthospital follow‐up prevents the assessment of functional sequelae. Furthermore, the single‐center design limits the generalizability of the results to other regions of the DRC.

### 4.2 Recommendations

The results call for:

1. The implementation of an effective prehospital evacuation system;
2. Strengthening surgical and resuscitation capacities in peripheral hospitals;
3. The development of standardized protocols for the management of open fractures and visceral injuries;
4. Increased coordination between health authorities and humanitarian actors.

## 5 Conclusion

This study showed that gunshot wounds in Goma primarily affect young men. Open fractures, abdominal injuries, hemodynamic instability, and prolonged delays to admission were some of the risk factors to be noted. The significant and primary causes of complications from gunshot wounds in Goma's conflict zone include wound contamination leading to infections, osteomyelitis, hemorrhagic shock from vascular damage, tissue cavitation causing compartment syndrome and fractures, organ perforation, and admission delays over 24 h correlating with fatal sepsis, exsanguination, multi‐organ failure, and even amputation. Strengthening the prehospital system, training staff, and implementing appropriate surgical protocols are essential to improving the prognosis for gunshot wound victims in conflict zones.

## Author Contributions

Conceptualization: Samuel Mbabazi and Zubayer Shams. Data curation: Samuel Mbabazi and Reagan Luvande. Formal analysis: Samuel Mbabazi, Aymar Akilimali, Zubayer Shams, and Reagan Luvande. Investigation: Samuel Mbabazi and Jones Onesime. Methodology: Samuel Mbabazi, Zubayer Shams, and Phalek Mukaka. Project administration: Samuel Mbabazi and Aymar Akilimali. Supervision: Médard Kabuyaya and Luc Kinyoma. Writing – original draft: All Authors. Writing – review and editing: All Authors. Final approval of manuscript: All Authors.

## Funding

The authors have nothing to report.

## Disclosure

All authors have read and approved the final version of the manuscript. Samuel Mbabazi and Médard Kabuyaya have full access to all of the data in this study and take complete responsibility for the integrity of the data and the accuracy of the data analysis.

## Ethics Statement

The protocol for this study was reviewed and approved by the Ethics Committee of the faculty of medicine of the University of Goma, DR Congo. This study was conducted in accordance with the ethical standards laid down in the 1964 Declaration of Helsinki and its later amendments or comparable ethical standards. Data were processed confidentially and anonymously.

## Consent

All participants were informed of the study objectives and written informed consent was obtained. We have taken steps to respect the dignity and anonymity of patients who participated in our study.

## Conflicts of Interest

The authors declare no conflicts of interest.

## Provenance and Peer Review

Not commissioned, externally peer reviewed.

## Transparency Statement

Samuel Mbabazi affirms that this manuscript is an honest, accurate, and transparent account of the study being reported; that no important aspects of the study have been omitted; and that any discrepancies from the study as planned have been explained.

## Data Availability

The authors have nothing to report.

## References

[1] Z. Ou , Y. Ren , and D. Duan , “Global Burden and Trends of Firearm Violence in 204 Countries/territories From 1990 to 2019,” Front Public Health 10 (2022): 966507, 10.3389/fpubh.2022.966507. 36111185 PMC9470124

[2] “Injury Prevention,” World Report on Violence and Health, accessed November 22, 2025, https://injuryprevention.bmj.com/content/9/1/93.1?int_source=trendmd&int_medium=cpc&int_campaign=usage‐042019.

[3] P. M. Budema , R. B. Murhega , and T. N. Tshimbombu , “Fatal and Nonfatal Firearm Injuries in the Eastern Democratic Republic of Congo: A Hospital‐Based Retrospective Descriptive Cohort Study Assessing Correlates of Adult Mortality,” BMC Emergency Medicine 21 (2021): 116, 10.1186/s12873-021-00506-3. 34641813 PMC8506075

[4] J. F. Bake , P. K. Mukama , J.‐P. MK , K. K. Medard , and M. A. Eugene , “Mortality of Trauma Patients in Conflict‐Affected Region: A Retrospective Observational Study of ICU Admissions and Surgical Management,” BMC Surgery [Electronic Resource] 25 (2025): 333, 10.1186/s12893-025-03090-6. 40751197 PMC12315310

[5] H. K. Bhattarai , S. Bhusal , F. Barone‐Adesi , and I. Hubloue , “Prehospital Emergency Care in Low‐ and Middle‐Income Countries: A Systematic Review,” Prehospital and Disaster Medicine 38, no. 4 (2023): 495–512, 10.1017/S1049023X23006088. 37492946 PMC10445116

[6] B. A. Solagberu , “Epidemiology and Outcome of Gunshot Injuries in a Civilian Population in West Africa,” European Journal of Trauma and Emergency Surgery 29, no. 2 (2003): 92–96, 10.1007/s00068-003-1285-5.

[7] M. Abghari , A. Monroy , S. Schubl , R. Davidovitch , and K. Egol , “Outcomes Following Low‐Energy Civilian Gunshot Wound Trauma to the Lower Extremities: Results of a Standard Protocol at an Urban Trauma Center,” Iowa Orthopaedic Journal 35 (2015): 65–69. 26361447 PMC4492129

[8] A. A. Asgedom , A. Etsedingl , and T. T. Hailemariam , “Prevalence, Causes and Outcomes of War‐Related Civilian Injuries in Ethiopia's War‐Torn Tigray Region: A Community‐Based Descriptive Study,” BMC Research Notes 16, no. 1 (2023): 352, 10.1186/s13104-023-06640-4. 38012754 PMC10683136

[9] V. Kapinga , D. Nsanduku , and B. K. Nkongolo , “Epidemio‐Clinical Profile and Management of Traumatic Injuries at the University Clinics of Kinshasa, DR Congo,” Journal of Global Health Economics and Policy 5 (2025): e2025033.

[10] L. M. Kalisya , J. F. Bake , and B. Elisee , “Surgical Repair of Orofacial Clefts in North Kivu Province of Eastern Democratic Republic of Congo (DRC),” Cleft Palate‐Craniofacial Journal 57, no. 11 (November 2020): 1314–1319, 10.1177/1055665620947604. 32787585

[11] P. B. Munguakonkwa , J. de Dieu Namegabe Tumsifu , and G. B. Murhula , “The Use of Negative Pressure Therapy for the Treatment of Gunshot Wounds in a Limited Resource Setting in Eastern Part of the Democratic Republic of Congo: Case Series,” Clinical Case Reports 12, no. 8 (August 2024): e9349, 10.1002/ccr3.9349. 39171333 PMC11335570

[12] H. H. Mohamed , H. A. A. Adan , S. Turfan , M. Aysin , and M. F. Y. Mohamud , “A Silent Epidemic: Exploring the Clinico‐Epidemiological Impact of Explosion and Gunshot Injuries in the Emergency Department of a Tertiary Hospital in Somalia,” African Journal of Emergency Medicine 15, no. 4 (December 2025): 100898, 10.1016/j.afjem.2025.100898. 40933061 PMC12419067

[13] T. N. Tshimbombu , M. N. Fefe , and M. Shin , “Demographic and Clinical Factors Affecting Pediatric Survival in South Kivu, the Democratic Republic of the Congo,” American Journal of Tropical Medicine and Hygiene 108, no. 1 (November 2022): 231–234, 10.4269/ajtmh.22-0455. 36410325 PMC9833088

[14] J. K. Mbeva , B. M. N. Vivalya , and J. K. Syayipuma , “Profile and Management of War Casualties in the Context of Limited Resources and Conflict in the Eastern Democratic Republic of Congo: A Retrospective Observational Study,” BMC Public Health [Electronic Resource] 26, no. 1 (November 2025): 48, 10.1186/s12889-025-25729-y. 41318421 PMC12771735

[15] K. Gumeniuk , I. A. Lurin , I. Tsema , L. Malynovska , M. Gorobeiko , and A. Dinets , “Gunshot Injury to the Colon by Expanding Bullets in Combat Patients Wounded in Hybrid Period of the Russian‐Ukrainian War During 2014–2020,” BMC Surgery [Electronic Resource] 23, no. 1 (January 2023): 23, 10.1186/s12893-023-01919-6. 36707838 PMC9883919

[16] P. S. Mudekereza , G. B. Murhula , and C. Kachungunu , “Factors Associated With Hospital Outcomes of Patients With Penetrating Craniocerebral Injuries in Armed Conflict Areas of the Democratic Republic of the Congo: A Retrospective Series,” BMC Emergency Medicine 21, no. 1 (October 2021): 109, 10.1186/s12873-021-00504-5. 34600474 PMC8487558

[17] K. Chrysou , G. Halat , B. Hoksch , R. A. Schmid , and G. J. Kocher , “Lessons From a Large Trauma Center: Impact of Blunt Chest Trauma in Polytrauma Patients‐Still a Relevant Problem?,” Scandinavian Journal of Trauma, Resuscitation and Emergency Medicine 25, no. 1 (April 2017): 42, 10.1186/s13049-017-0384-y. 28427480 PMC5399315

[18] J. Doran , M. Salih , and A. Bell , “Major Trauma Patients and Their Outcomes—A Retrospective Observational Study of Critical Care Trauma Admissions to a Trauma Unit With Special Services,” Injury 55, no. 8 (August 2024): 111622, 10.1016/j.injury.2024.111622. 38905903

[19] P. M. Budema , R. B. Murhega , and T. N. Tshimbombu , “Hand Grenade Blast Injuries in the Eastern Democratic Republic of Congo: A Case Series of 38 Patients,” BMC Emergency Medicine 22, no. 1 (March 2022): 43, 10.1186/s12873-022-00599-4. 35305564 PMC8933972

